# Notes

**Published:** 1878-07

**Authors:** 


BRAIN: A JOURNAL OF NEUROLOGY.—Edited by Drs. Bucknill, Crichton-Browne, Ferrier, and Hughlings-Jackson, and published by Messrs. McMillan & Co., London and New York. The Editors of "Brain" make no apology for adding another to the already long list of British scientific journals. The want has been long felt of a periodical devoted to the discussion of kindred subjects, not yet adequately represented in existing medical and scientific serial literature.

It will be the object of "Brain" to keep its readers well abreast of modern progress in Neurology, and to advance the knowledge of a class of diseases respecting which, it is universally admitted that much has yet to be learned.

THE AMERICAN SERIES OF CLINICAL LECTURES.—With the issue of No. 36 of the Series of Clinical Lectures, the third volume will be completed, and the publication of the Series will for the present be discontinued.

The three volumes now completed contain an exceptionally valuable series of monographs on practical subjects, by some of the ablest instructors in the profession, and the publishers expect to obtain for them (at the reduced price of $3) a permanent sale.

Single numbers of all Lectures can also be obtained at the price of 25 cents each.

